# A prospective observational study of decision‐making by patients with amyotrophic lateral sclerosis upon recommendation for PEG enteral feeding tubes

**DOI:** 10.1002/ncp.11290

**Published:** 2025-03-18

**Authors:** Kay Tran, Heather A. Hayes, Mark Bromberg

**Affiliations:** ^1^ Department of Neurology University of Utah Salt Lake City Utah USA; ^2^ Department of Physical Therapy and Athletic Training University of Utah Salt Lake City Utah USA

**Keywords:** ALS, amyotrophic lateral sclerosis, decision‐making, feeding tube, gastrostomy, PEG, percutaneous endoscopic gastrostomy

## Abstract

**Objective:**

To understand challenges surrounding acceptance of a percutaneous endoscopic gastroscopic enteral feeding tube by patients with amyotrophic lateral sclerosis: a prospective observational study.

**Methods:**

This was a prospective observational study of 41 patients and care partners attending a multidisciplinary Motor Neuron Disease clinic. Surveys were administered pregastrostomy tube placement (*N* = 23) and postplacement (*N* = 41). Some were not available both pre‐ and postplacement). For preplacement, we queried barriers affecting their decision for receiving a gastrostomy tube at the time of recommendation. For postplacement, we queried factors that influenced their decision as well as perceived benefit and satisfaction with use.

**Results:**

Patient concerns about receiving a gastrostomy tube centered on the procedure, possible pain/infection (48%), limitations on activities (44%), impact on body image, and possible extension of life. For patients who received a gastrostomy tube, satisfaction was very high (93%), and there was reduced patient (59%) and care partners (54%) stress. The average BMI was 28.6 kg/m^2^ at diagnosis, and there was no net gain in weight. The average time until placement of a gastrostomy tube following recommendation was 145 days (range 13–824 days).

**Conclusions:**

Despite counseling at multiple time points, the decision to obtain a feeding tube is often challenging for patients and care partners. Gastrostomy tube placement was perceived as a substantial benefit. Addressing these barriers may reduce concerns and promote earlier decision‐making to maximize the benefits of placing a gastrostomy tube sooner.

## INTRODUCTION

Weight loss frequently occurs in patients with amyotrophic lateral sclerosis (ALS).^1^ Common motoric reasons include dysphagia and arm/hand weakness affecting food delivery.^2^ Metabolic factors are higher energy expenditure requirements with increased respiratory effort, muscle fasciculations, and, in some, hypermetabolism.^3^, ^4^ Other factors are dehydration, decreased food intake caused by a loss of appetite, hypogeusia, fatigue, depression, inadequate caloric intake, and constipation due to decreased physical activity and medications.^5^, ^6^

Guidelines for nutrition management recommend a gastrostomy feeding tube (G‐tube) when there is a 10% weight loss over 6 months or a progressive loss of weight despite nutrition supplements.^7^ There is a statistical association between increased survival among patients with ALS with a premorbid body mass index >30 kg/m^2^; therefore, early gastrostomy tube placement, before significant weight loss, is felt to be advantageous.^1^, ^8^ The reasons for this association are not clear, but a role of fat metabolism is implicated.^1^ Severe dysphagia can hamper taking medications, which can be delivered more easily (crushed or in liquid form) through a feeding tube. Other advantages include fewer choking episodes while trying to eat a full meal and maintain adequate fluid intake, with reduced risk of aspiration and potential respiratory infections.^9^ Gastrostomy tube placement is considered advantageous for the care partner and family because there is a reduced fear of choking episodes and reduced frustration with efforts to make meals enticing for patients. In contrast, weight loss may be viewed favorably by patients as loss of adipose stores can represent success after a long‐time struggle being overweight. Patients do not appreciate that weight loss draws from both adipose tissue and protein stores, some of which includes muscle protein.^10^

The decision to obtain a gastrostomy tube is often challenging for patients and care partners.^11^ Reluctance continues despite detailed education about advantages, the placement process, relative invisibility in usual attire, and the ability to still take bites and sips for pleasure. To better understand challenges surrounding acceptance of a gastrostomy tube, we queried patients about barriers and factors affecting their decision for receiving a gastrostomy tube, and among those accepting a gastrostomy tube we also queried perceived benefits and satisfaction with the use of the gastrostomy tube. Finally, we sought to understand the timeframe from clinical recommendation to receiving a gastrostomy tube and the percent weight loss that occurred before and after gastrostomy tube placement.

Educational efforts likely vary among providers even at multidisciplinary clinics. ALS care guidelines do not comment on how best to present enteral nutrition to prospective patients.^12^ Educational formats include internet descriptions and videos and publications (books), but their efficacy has not been assessed.

## MATERIALS AND METHODS

### Study design

This was a prospective, longitudinal study performed from March 2022 to October 2023 on individuals attending the University of Utah multidisciplinary Motor Neuron Disease clinic, Salt Lake City, UT. Patients who met our criteria for gastrostomy tube placement (>10% weight loss based on weight at time of diagnosis) were queried with surveys during their routine 3‐month interval clinic visits. This study was approved by the University of Utah Institutional Review Board, with an exempt status. No identifying information was collected on the patients.

### Participants

At each visit swallowing and nutrition evaluations were made by the registered dietitian (Author KT), speech‐language pathologist, and neurologist. When all three providers agreed that a gastrostomy tube would be warranted, patients were verbally educated about the goals and benefits of a gastrostomy tube. Patients also received information on the benefits of a gastrostomy tube at the initial clinic visit presented as a chapter in a book with photographs showing the invisibility of the tube under clothes and a discussion on the ability to continue eating. The pre–gastrostomy tube placement survey was administered immediately after education and discussion in clinic.

All patients received gastrostomy tube by percutaneous endoscopy (PEG) by a gastroenterologist with a tube of their choice. Noninvasive ventilation was used at the discretion of the gastroenterologist. Patients received instructions for use by a home health visiting nurse 24 h after placement. Nutrition needs were determined before placement and adjusted as needed.

### Surveys

The pre–gastrostomy tube survey focused on nine potential barriers: (1) concern about the procedure, (2) appearance of the device [visible to others], (3) change in body image, (4) limitations on activities, (5) difficulty with use, (6) limitations on ability to eat and drink by mouth, (7) whether it will extend their life, (8) extra expense, (9) risk of infection or pain, and (10) other reasons not specifically queried could be written in. We asked individuals about what terminology was preferred when discussing a feeding tube, and we sought to understand any misconceptions about a gastrostomy tube and a nasogastric tube (NG).

The post‐gastrostomy tube survey asked about: (1) factors that influenced their decision ([a] reducing anxiety, [b] improving hydration, [c] extending life, [d] reducing care partners stress, [e] reducing weight loss, [f] reduce choking/swallowing, [g] clinic team recommendation, [h] intubation [tracheostomy plus gastrostomy tube]); (2) satisfaction with the feeding tube ([a] overall satisfaction, [b] would they do the procedure again, [c] would they recommend the procedure to other patients, [d] was it easier or as expected to use, [e] was it harder to use than expected, [f] was there reduced stress for the patient, [g] was there reduced stress for the care partners, and [h] did the gastrostomy tube improve the closeness between the care partners and patient); and (3) benefits of receiving the gastrostomy tube ([a] greater energy, [b] ability to maintain weight, [c] less time spent eating, [d] improved quality of life, [e] less stress, [f] ease of medication administration, and [g] improved hydration).

Survey responses were recorded on paper, and patients could choose all answers that applied. Counts for each answer were recorded and tallied. Individuals and care partners both participated in these surveys. Email correspondence was not utilized and there were no refusals to participate.

### Additional variables

The timeframe that recommendations were made (based on 3‐month clinic visits) and the time the feeding tube placement took place (based on procedure date in the chart) were recorded. BMI and change in weight (percent) from time of diagnosis to time gastrostomy tube was first recommended, and change in weight after receiving gastrostomy tube were calculated.

### Analysis

This was an observational study with relatively small sample size, and statistical analyses were not performed. Response counts for each question were recorded and tallied and a percentage reported. The timeframes for recommendation and placement of the feeding tube were calculated as central tendency in days.

## RESULTS

### Participants

Fifty‐one individuals participated; 49 had a diagnosis of ALS and two had bulboamyotrophy or Kennedy's disease, as they have similar issues with bulbar weakness and weight loss. Pre–gastrostomy tube placement: 23 individuals were queried when gastrostomy tube placement was clinically indicated (pregastrostomy), and 16 individuals completed both pre‐ and post‐gastrostomy tube surveys. Two individuals who completed pre‐gastrostomy tube surveys chose not to pursue placement during the time frame of the study. For seven, data were incomplete for different surveys, and for some assessment of satisfaction was missing. Post–gastrostomy tube placement: 41 individuals were queried, 16 of which completed both pre– and post–gastrostomy tube surveys. Several died before receiving a gastrostomy tube. Consequently, as this was a prospective study, we included 24 individuals who were not queried before placement during the study period. Not all questions in the surveys were addressed and denominators vary in number.

Participant ages were from 24 to 83 years with an average age of 58.2 years. There were 29 men and 22 women The average BMI was 28.6 kg/m^2^ at diagnosis. At the time of recommendation for gastrostomy tube placement ALS Functional Rating Score‐Revised Swallowing sub score average was 1.68, and the average combined Speech, Salivation, Swallowing score was 6.32.

### Pre‐gastrostomy tube placement

**Figure 1:**
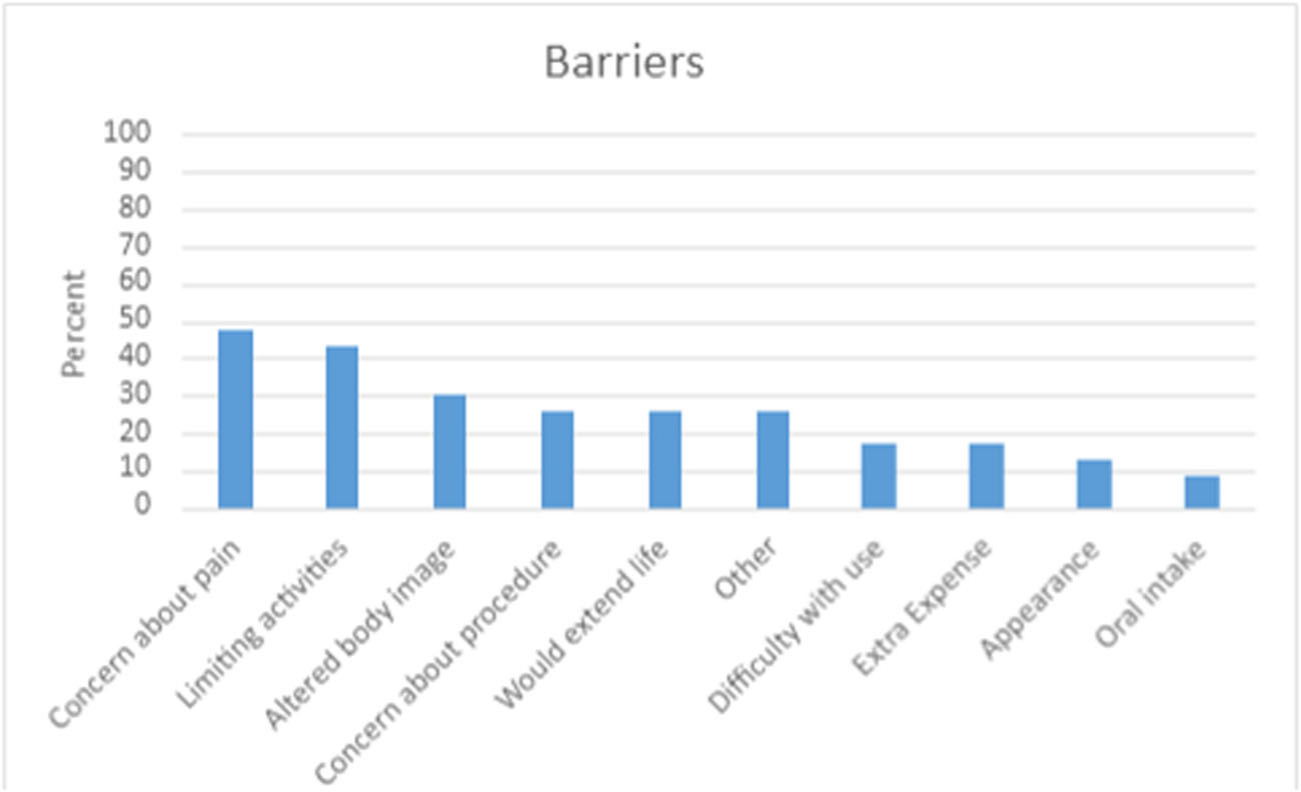
Assessment of potential barriers identified by patients before receiving a gastrostomy tube.

The most cited barriers were concerns about pain or infection (47.8%; 11/23) and limitation of activities (43.5%; 10/23) (Figure 1). Less common barriers were impact on body image (30.0%; 7/23), concern about the procedure (26.0%; 6/23), and extension of life (26.0%; 6/23).

There was no conclusive preference for terminology when discussing a feeding tube, with equal preference for gastric feeding tube (30%; 7/23), alternate method of nutrition (26%; 6/23), gastrostomy tube (22%; 5/23), and tube feeding (22%; 5/23).

Fifty‐nine percent of participants knew the difference between a nasogastric (NG) feeding tube and a gastrostomy feeding tube. Additionally, written responses suggested a negative opinion of NG tubes as NG tubes looked unattractive and uncomfortable. Many participants did not realize that eating and drinking by mouth is an option even after gastrostomy tube placement (17%; 6/36).

### Post–gastrostomy tube placement

**Figure 2:**
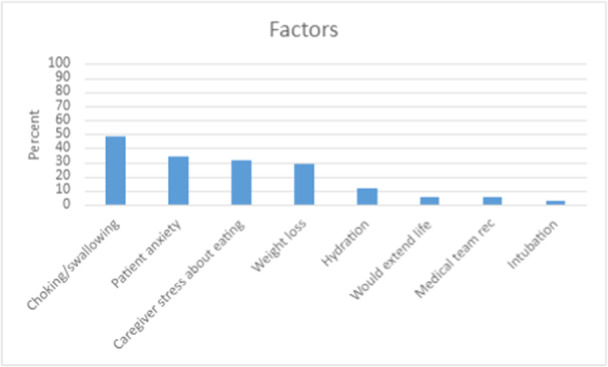
Assessment of factors that have influenced individuals' decision‐making after receiving a gastrostomy tube.

**Figure 3:**
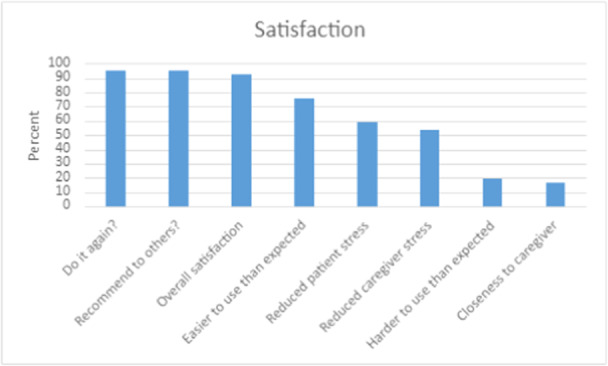
Satisfaction with their decision after receiving a gastrostomy tube.

Factors influencing the patient's decision about obtaining a gastrostomy tube focused on perceived positive outcomes, including reduction of choking and swallowing (48.6%; 17/35), reduced patient anxiety (34.0%; 12/35), reduced care partners stress (31.4%; 11/35), and ability to maintain weight (28.6%; 10/35) (Figure 2). Patients who received a gastrostomy tube were assessed on satisfaction with the decision (Figure 3), and overall satisfaction was high at 92.5% (37/40). Additionally, the gastrostomy tube was easier to use or was as expected to use (75.6%; 31/41) and resulted in reduced patient stress (58.5%; 24/41) and reduced care partners stress (53.7%; 22/41).

**Figure 4:**
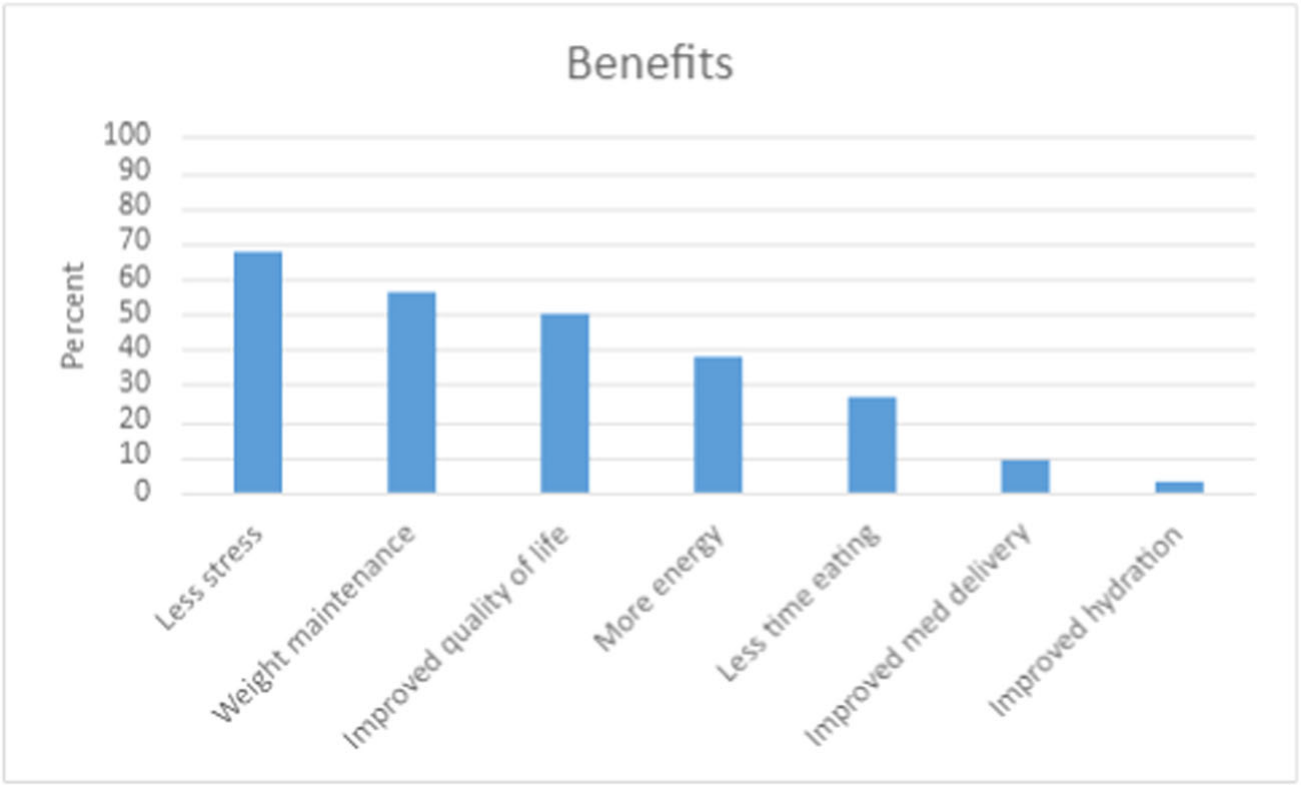
Benefits gained from their decision to receive a gastrostomy tube.

Patients were also assessed on perceived benefits (Figure 4). The primary benefit was less stress around eating (67.7%; 23/34), ability to maintain weight (55.9%; 19/34), quality of life (50.0%; 17/34), and improved energy (38.0%; 13/34).

### Additional variables

Recommendations for gastrostomy tube placement were made an average of 639 days (range 14–824 days; SD ± 710) after date of diagnosis. The time to receive a gastrostomy tube from time of recommendation was a mean 145 days (range 13–824 days; SD ± 184).

The average percent weight loss from diagnosis to the recommendation for a gastrostomy tube was 6% (range from −21% loss to 7% gain). Despite gastrostomy tube placement individuals continued to lose weight to the end of the study (mean 3% loss; range −19% loss to 11% gain).

## DISCUSSION

When a feeding tube is felt to be beneficial for a patient with ALS, there are challenges in how to present both the procedure and benefits. We found that many patients are reluctant to proceed because the mean time to receive a gastrostomy tube placement from recommendation was 145 days (range: 13–824). In this study we sought to identify barriers and factors influencing their decision and common misconceptions which may be held, with the goal of modifying our approach in educating patients to help with the process. We also queried individuals who received a feeding tube for perceived benefit and satisfaction.

Although our study queried a relatively small number of patients, the survey results are largely in line with our subjective impression, and we feel they are generally applicable. Before our study, during our conversations with patients and care partners when a recommendation was made, we frequently encountered several misunderstandings, including perceived pain or infection, limitations of activities, alteration of body image, concern about the procedure, and interestingly, possible extension of life, and these issues were distributed with similar percentages (range 26%–47%). These misunderstandings led to our study. Compared with the initial misunderstandings and misgivings, we found a very high degree of satisfaction after placement (92.5%). Most patients found the gastrostomy tube was easier to use than expected (75.6%), and, importantly, half noted reduced patient and care partners' stress. An interesting concern raised in 26% of patients is that a feeding tube could extend life. Data are not clear whether survival is predictably prolonged with a feeding tube because some studies show no prolongation and others prolongation.^13^, ^14^ Patient perception of how life will be at late stages is difficult to predict, and studies indicate many factors involved but enteral feeding does not have a negative impact on patient mood.^15^ How the concern over enhanced longevity fits into other patient choices, such as whether to take disease modifying drugs for ALS that have been shown to extend survival, is not clear but deserves more study. An associated factor is the burden on the care partners to manage tube feedings. We anticipated that the addition of the feeding tube for nutrition would lead to an overall weight gain, as some studies have suggested.^1^, ^13^, ^16^ However, data are conflicting because it has been suggested that gastrostomy tubes are commonly inserted too late during the disease course, minimizing expected weight gain.^17^ Our study confirmed both a delay in placing the gastrostomy tube based on our time of recommendation and placement (approximately 5 months) and general lack of weight gain. We would like to point out that placement of a tube does not guarantee utilization of the tube by the patient or taking the recommended amount of formula, and utilization of the tube should be included in a follow‐up study.

In our clinic, we include discussion and written information on benefits of a feeding tube at the initial visit and at time of recommendation. Overall, our study shows that despite these efforts providers can do more in the presentation of a gastric feeding tube to correct misunderstandings. What to do and how to do so are unclear. Showing a video which both explains and shows use of the feeding tube would supplement a discussion; we now have computers in our clinic with the ability to show a video at the time of recommendation. A supplement to the video with captions or patient and care partners offering their experiences would be helpful. Many multidisciplinary clinics have support groups and presentations by patients and care partners and could describe benefits and field questions. Data on extending life with use of enteral feeding are equivocal, and given the mixed feelings expressed by patients, these effects should receive less emphasis.

We plan a follow‐up study when our approach has been modified with respect to the above additions.

## LIMITATIONS

The limitations of our study were the relatively small sample size, missing data, and not all participants responding to all the questions consistently.

## CONCLUSION

In summary, we have identified patient concerns for agreeing to a gastrostomy tube and include a spectrum of formidable barriers. With these data in mind, we propose changes to presenting gastrostomy tube nutrition.

## AUTHOR CONTRIBUTIONS

Kay Tran, Mark Bromberg, and Heather Hayes conceived and designed the analysis.; Kay Tran collected the data.; and Kay Tran, Mark Bromberg, and Heather Hayes wrote the paper.

## CONFLICT OF INTEREST STATEMENT

None declared.
